# Caregiving Across International Borders: a Systematic Review of Literature on Transnational Carer-Employees

**DOI:** 10.1007/s10823-022-09468-w

**Published:** 2022-12-19

**Authors:** Bharati Sethi, Allison Williams, Joyce L. S. Leung

**Affiliations:** 1 Department of Political Studies, Champlain College, Trent University, CCW 1 1600 W Bank Drive, Peterborough, ON K9L 0G2 Canada; 2 School of Earth, Environment & Society, McMaster University, 1280 Main St West, Hamilton, ON L8S 4L8 Canada; 3 School of Social Work, King’s University College, 266 Epworth Ave, N6A 2M3 London, ON Canada

**Keywords:** Immigration, Transnational caregiving, Transnational carer-employees, Global, Ageing

## Abstract

In diaspora and transnational studies little is known about the experiences of transnational carer-employees (TCEs). TCEs provide unpaid/informal care across international borders to an adult family member, friend, or relative with disability and/or age-related needs, while also working in paid employment in the country of resettlement. The primary focus of this systematic review was to examine how cultural and historical elements of transnational caregiving influence the economic, social, and health/well-being of TCEs. This systematic review draws on quantitative and qualitative peer-reviewed literature on TCEs’ experiences from Canada, the USA, or Australia between 1997 and 2017. In all, 16 articles that fulfilled the search inclusion criteria were selected. The articles were analyzed using content and thematic analysis. The review highlighted that transnational caregiving is a multi-dimensional phenomenon. There is a reciprocal relationship between adult children providing care to their parents and parents helping their children resettle in their new home. The findings suggest that TCEs provide practical, financial, and emotional care to their families abroad. To the best of our knowledge, this is the first comprehensive systematic review of the experiences of TCEs. Increasingly complex immigration experiences of transnational families require innovative policy responses from a transnational and intersectionality lens. Immigrants need support to maintain solid transnational networks and simultaneously adapt to the country of resettlement. Employers can use the findings to support TCEs in balancing unpaid care across vast geographical distances while sustaining their economic and social well-being.

## Introduction

The past century has been marked by a rise in the world’s migrant population and increased discussions about globalization and transnationalism (Bernhard et al., 2008; Tung, 2000). In 2017, Immigration, Refugees and Citizenship Canada (IRCC) admitted 22,253 caregivers as permanent residents into Canada (Government of Canada, 2019). Two characteristics inform these immigration trends. First, in recent years, the proportion of immigrants from Europe, the United States of America (USA), and the United Kingdom has decreased. In contrast, the proportion of recently arrived immigrants from South Asia has steadily increased (Edmonston, 2016). It is important to note that in almost every country in the South Asian region, the provision of care to ageing parents is a primary responsibility of the children, especially in Muslim countries where it is considered a religious duty. Second, Canadian institutions have been promoting the Canadian education system across borders since 2000 and have attracted almost half a million international students from all over the world. According to the Canadian Bureau for International Education (CBIE), the total number of international students in Canada has increased by 119% between 2010 and 2017 (CBIE, 2018). According to recent statistics, 642,000 international students are enrolled in all levels of study across the globe; with respect to the hosting countries with the largest number of students, Canada is ranked third, after the United States of America and Australia (El-Assal, 2020). CBIE reports that 51% of these international students plan to apply for permanent residency in Canada. Interestingly, more than 50% of all international students come from China (28%) and India (25%), where the notion of providing care to ageing parents is considered a “normative obligation” (Baldassar et al., 2007, p. 15). These new immigration trends and increasingly complex immigration experiences of transnational families urge us to explore and study this emerging phenomenon of transnational caregiving within the Canadian context.

The provision of family caregiving to parents is a deeply rooted tradition in many societies, particularly in Asian and African countries (Dhar, 2011a; Kodwo-Nyameazea & Nguyen, 2008). This essential cultural practice has been affected by international immigration to developed countries. The result of this migration trend is the formation of transnational families separated by country of origin and country of employment (Lahaie et al., 2009). The existing literature on transnational caregiving indicates that despite all its complications due to geographic distance, the concept of family caregiving has not lost its importance within families. Transnational family members manage to maintain ties to their home country and provide care to their family from a distance (Baldock et al., 2007, as cited in Amin & Ingman, 2014). In their study with Ghanaian-Akan transnational caregivers in the USA, Kodwo-Nyameazea & Nguyen (2008, p. 288) observed that some participants considered elder caregiving a “religious” or a “moral” obligation. Yet, for other participants, the older adult’s “life ‘depend upon it’” (Kodwo-Nyameazea & Nguyen, 2008, p. 288). As such, different dimensions and strategies of family caregiving are developed (Dhar, 2011a, b), and new challenges and dilemmas are experienced by members of transnational families (Gui & Koropeckyj-Cox, 2016; Baldassar et al., 2007).

In the age of the proliferation of media platforms and the affordability of communication technologies, Madianou (2014) proposes the theory of polymedia to unpack the complexity of transnational communications. While new media (such as WhatsApp and Zoom) provide opportunities for frequent transnational communication, the author argues that immigrants intentionally choose a specific media platform to manage, negotiate, and mediate their relationships across international borders (Madianou, 2014). His work with Philippine mothers suggests that new media and communication platforms are “not the only burden of communication: women reported that the requests for help intensified putting a strain on their resources. Crucially, every mediated interaction is a reminder of the distance involved and the fact that ‘you can’t hug your children” (Madianou, 2014, p. 337). Dekker and Engbersen’s work with international migrants reveals that social media facilitates and sustains strong long-distance ties and strengthens weak ties (2014). The open structure of media offers migrants opportunities to create “communities of choice based on interests rather than prior acquaintance,” thus, activating “latent ties” (Dekker & Engbersen, 2014, p. 404). The COVID-19 pandemic highlighted the importance of virtual platforms, including Facebook, WhatsApp and Zoom, used by immigrants to sustain transnational ties (Sethi, 2022).

Due to technological advances “ageing parents in Italy could experience co-presence or ‘real time’ interactions” with their grandchildren in Australia (Miyawaki & Hooyman, 2021, p. 13). Despite the choices and affordability of social media, these virtual networks and communication resources are inaccessible to some migrants based on their socioeconomic status, gender, age, education levels, and geography (Dekker & Engbersen, 2014; Madianou, 2014). Further, the use of technology is dependent upon its’ cost and accessibility within the country of migration and resettlement (Miyawaki & Hooyman, 2021). Another important point to consider is the fact that although technology “can increase feelings of psychological closeness, it does not completely eliminate the effects of geographic distance” (Wilding, 2006, as cited in Miyawaki & Hooyman 2021, p. 13). Participants in Sethi’s (2022) study with European transnational caregivers found that they missed the “physical/human” touch (p. 4) as they were unable to travel home due to COVID-19 pandemic-related travel restrictions. Such “virtual co-presence” does not satisfy immigrants’ longing to be physically present with family members (Baldassar, 2008, p. 252). Therefore, although migration across national borders is not a new occurrence (Tung, 2000), the experience of global family care and the complex and diverse ways transnational families manage long-distance relationships prompts researchers to study this phenomenon.

Given the increase in immigration and ageing demographics, studies on transnational caregiving have rapidly increased in the last decade. Scholars have provided valuable insights into the dynamics of transnational family networks. Brijnath (2009) uses her personal experience as a transnational caregiver living in Australia and providing care to her grandmother with severe dementia in India, to advance our understanding of the social and emotional networks within transnational families, as well as the power dynamics that underlie transnational caregiving transactions. Baldassar et al., (2007) have completed the most comprehensive research on transnational caregiving and define transnational caregiving as “the exchange of support and care across distance and national borders” (p. 26). Nevertheless, only informal caregiving (predominantly emotional and moral support) within transnational families (e.g., Baldassar 2007a, b, 2011, 2013, 2016; Baldassar et al., 2016, 2017; Brijnath, 2009; Dekker & Engbersen, 2014; Madianou 2014, 2016; Madianou & Miller, 2013; Spitzer et al., 2006) have been studied. Others have focused on the experiences of formal/paid immigrant carers in their country of resettlement (e.g., Donovan & Williams, 2014; Ho et al., 2003; Ilegan et al., 2020; Lai, 2007, 2008; Lai & Leonenko, 2007; Park, 2012; Turcotte & Savage, 2020). Except for scant literature (Sethi, 2022; Sethi & Williams, 2016, 2017; Sethi et al., 2017a, b, 2018), research that exclusively focuses on the experiences of transnational carer-employees is absent.

Carer-employees (CEs) are defined as “family members and other significant people, who provide care and assistance to individuals (i.e., parent, spouse or life partner, adult child, sibling and/or friend) living with debilitating physical, mental and/or cognitive conditions, while also working in paid employment” (Ireson et al., 2018, p. 1–2). Given the increased migration of skilled labour from developing countries to Canada, it is surprising to note that at the time of the systematic review, there were no research studies, to our knowledge, that exclusively focuses on transnational carer-employees (TCEs) in Canada; thus, providing the impetus for this study. TCEs are carer-employees who work in paid employment in Canada and provide familial care in their country of origin. Different terms for these carers are used in the literature, including transnational carer-employees, transnational caregiving employees, and caregiver-employees (Ireson et al., 2018; Lorenz et al., 2021).

It is noteworthy that even after 2017 (our review focused on peer-reviewed literature published prior to 2017), there is a paucity of research on TCEs. For example, in a recent transnational caregiving systematic review, Miyawaki and Hooyman (2021) addressed the gap in the literature on transnational caregiving experiences and characteristics of immigrant adult children to ageing parents. While their review highlighted national and employer policies as an important political context that impacts adult children’s ability/inability to provide transnational care, the study was not focused on TCEs. These authors made an important observation that workplace policies and programs such as flexible family leave would help immigrants make extended visits to their ageing parents/families. Earlier research suggests that lack of workplace support via caregiver-friendly workplace practices (CFWPs) can result in caregiver-employees (CEs) leaving the workforce, and/or missed workdays, early retirements, reduced productivity, health consequences and increased avoidable costs to employers (Ireson et al., 2018; Vuksan et al., 2012a, b; Lorenz et al., 2021).

## Current Study

Our research builds on current caregiving literature by situating carer-employees experiences with a transnational migration context. Transnational caregiving is emerging as a new field of research in the social sciences, specifically within family and migration studies. Specifically, we conducted a systematic review^1^ of the experiences of TCEs in Canada, Australia, and the USA. The primary focus of this systematic review was to examine how cultural and historical elements of transnational caregiving influence the economic, social, educational, and health well-being of TCEs.

Another goal of the review was to gather available evidence to inform caregiver-friendly workplace practices (CFWPs). The realization of CFWPs in Canadian workplaces will be informed by the creation of a bilingual and culturally informed ‘Carer-Inclusive and Accommodating Organizations Standard’ and accompanying ‘Implementation Guide’^2^ (Canadian Standard Association, 2018). The ‘Carer-Inclusive and Accommodating Organizations Standard’ is a set of guidelines that can be used by employers, human resource professionals, labour organizations, and employed carers to meet legal requirements and help enhance work-life balance, improve workforce retention, and reduce healthcare costs (Williams et al., 2018). The accompanying ‘Implementation Guide’ is a practical handbook that provides step-by-step instructions on how to create CFWPs.

The overarching research question guiding this study was: *What is the significance (economic, social, and health/well-being) of transnational caregiving on TCEs residing in Canada, Australia, and the USA?* Our review explored the following sub-questions:

- What are the common patterns and practices of transnational caregiving? In what ways do TCEs/transnational families use to provide caregiving to their families who are left behind in their home countries?
- What are the challenges and dilemmas of TCEs/transnational families? What are the consequences of these challenges and dilemmas?
- Which cultural-historical elements influence the notion of transnational caregiving?

## Research Methodology

A systematic review was conducted to identify and summarize the available empirical evidence on TCEs’ experiences of providing transnational caregiving to their ageing parents, adult children, siblings and/or friends. This review was conducted by employing the systemic strategy of literature review, which is “a step by step process that is followed in systematic review” (Benach et al., 2010 as cited in Sethi, 2014, p. 21).

### Search strategy and data sources

The search was conducted over one month in 2017, beginning in September 2017. Original peer-reviewed articles of “Transnational Caregiving Employees in Canada” published in English since 1989 were searched beyond King’s University College, Western University’s library collection across all subject areas. We chose to review studies from 1989 to 2017 to span the most recent 28-year publication period in caregiving research within the North American context. We decided on this period because the Canadian immigration policy introduced significant changes during this time frame, promoting “an expanded proliferation of transnational family arrangements” (Bernhard et al., 2008, p. 4). For example, in 2002, the Canadian government introduced the Immigration and Refugee Protection Act (IRPA) and removed the Immigration Act of 1978 (Bernhard et al., 2008). Changes in immigration policies promoted the reunification of families and embraced multiculturalism, aiding the entry of migrants from non-European countries to North America (Bernhard et al., 2008).

### Inclusion criteria

Initially, all peer-reviewed studies selected for inclusion comprised of caregiving experiences of transnational families providing care from Canada to their ageing parents, adults living with disabilities, or children residing in their home country. The key search term “Transnational Caregiving Employees” was used together with “Canada.” Due to the lack of literature on this in the Canadian context topic, a criterion for inclusion was developed to refine the search strategy and identify the inclusion and exclusion of retrieved literature for the final selection of papers. Specifically, the inclusion criteria were expanded to include peer-reviewed literature on TCEs’ caregiving experiences produced in Australia and the USA.

We included Australia due to similar immigration laws, policies, and migration patterns compared to North America. For example, Australia and Canada are the two largest immigrant-receiving countries globally on a per capita basis (Clarke & Skuterud, 2013). Both governments adopted a points system in the 1970s, producing diverse immigrants from Asia, Africa, and the Middle East (Clarke & Skuterud, 2013).

### Exclusion criteria

1. While dissertations, conference papers, conference abstracts, books and book chapters are scholarly, they generally do not go through a peer-review process;
2. Grey literature data such as “print or electronic information not controlled by commercial or academic publishers including non-indexed conference abstracts frequently published in journal collections, dissertations, press releases, government reports, policy documents, book chapters or data obtained from trial registers” (Schmucker et al., 2017, p.2);
3. Articles on paid care workers under the Live-in-Care program (this program no longer exists in Canada) and temporary Foreign Worker Program (TFWP) as these programs adhere to different immigration policies and work permits than the TCE population.

### Article Selection

The article selection was complete in two phases:

### Phase One: Scanning and Surveying the Literature

This search involved two steps. In step one, the Research Assistant (RA) retrieved publication using the Summon search engine that permits access to thousands of journal articles indexed in over 700 indexes and databases in a single search. In step two, she searched the relevant literature in 13 individual databases.

#### Step One

Publication was retrieved through an electronic search using Summon for peer-reviewed academic and non-refereed articles.

##### First Screening

The RA did a broad search of “Transnational Caregiving employees” in Summon. She used several search words in various combinations with *Transnational Caregiving Employees (TCEs).* For example, she combined *Transnational Caregiving Employees (TCEs)* with “transnational caregiving employees,” “transnational care-giving employees,” “transnational caregiving.” As such, with AND intersectionality, she combined *Transnational Caregiving Employees (TCEs)* with two key search terms, “ageing population” and “Canada.” In the first screening, she also used the following phrase, *“Immigrant employees in Canada provide caregiving to families living across the border.”*

Realizing the paucity of literature on TCEs in Canada, the research team consisting of the RA, first author (A1) and second author (A2) extended the location of research studies to the USA and Australia. The RA used the search terms in combination (Transnational Caregiving Employees) AND (Canada) AND (USA) AND (Australia) to include these two countries.

The first screening produced **3793** search results that included 3433 Book/eBook, 156 Book Chapters, 16 Book Reviews, 133 Journal Articles, 29 Dissertations, 3 Newspaper Articles, 1 Publication, 9 References, and 1 Trade Publication Article. (See Table 1). Only the topics and abstracts of **133** journal articles were reviewed as systematic reviews are concerned with assessing the quality of included studies rather than examining the magnitude of the literature available around a specific topic (Arksey & O’Malley, 2005). We reviewed the papers that addressed some aspects of the research questions outlined earlier. Several research papers on the caregiving experiences of transnational families in Australia and the USA were rejected as not relevant to the inclusion criteria.

**Table 1:** First screening on Summon (Beyond King’s University College, Western Library Collection Across All Subject Areas)

Key Search Terms	Total Search Results	Journal Articles
(Transnational Caregiving Employees)	4370	303
(Transnational Caregiving Employees) AND (ageing population) AND (Canada)	2994	51
(Transnational Caregiving Employees) AND (Canada)	3793	133

##### Second Screening

The RA and the first author (A1) skimmed through the topics and abstracts of 133 articles. Articles that were not relevant to the topic were eliminated, such as the articles related to the Live-in-Care program and migrant farmworkers. Others discussed multi-level policy approaches in the governance of labour migration in the global-care chain system, temporary foreign worker programs in Canada, and employee-work-family relationships in a global context. This reduced the number of articles to **44.**

##### Third Screening

The RA downloaded and transferred 44 articles to Mendeley reference management software during this screening process. Information such as the author’s name, the title, the year of publication, and the journal the article was published in, was entered. The selected articles were shared with the research team for review and rating purposes.

The research team consisting of RA and the first two authors (A1 and A2) read through the full text of the **44** articles. For this final screening process, we adapted Westhues et al.’s (2008) evaluation criteria of quality assessment. The research team used a Likert-type scale to rate articles based on their relevance to the topic. Based on the evaluation criteria of Westhues et al. (2008), the 44 articles were ranked using a 5-star scale, ranging from not relevant, [given one star (*)] to highly relevant [given five stars (*****)], based on providing information on TCEs in Canada. Three- to five-star articles were automatically included in the final selection of the studies for the review, as they were based on transnational caregiving in Canada. However, two-star articles were also included in the review due to the absence of articles written on this topic in the Canadian context. The one-star articles were excluded. Once the research team rated the **44** articles, and after a collaborative discussion between the team members, **8** articles were included in the review. (See Table 2).

**Table 2:** Article selection in step 1 and step 2

Selection criteria	Likert Scale	STEP 1: # of Selected Journal Articles in Summon	STEP 2: # of Selected Journal Articles in Databases
(Transnational Caregiving Employees) AND (Canada) AND (Ageing Population)	*****	1	0
(Transnational Caregiving Employees) AND (Canada) AND (Adult living with a Disability)	****	0	0
(Transnational Caregiving Employees) AND (Canada) AND (Children)	***	1	0
(Transnational Caregiving Employees) AND (Canada) AND (USA) AND (Australia)	**	6	8
Total Articles Selected		**8**	**8**

#### Step Two

As the research team wanted to ensure they did not miss any literature on TCEs in Canada, the RA searched the relevant databases individually. The research team used the same selection process as explained in step one. She also consulted with the King’s University College, Western University, “to ensure that the search was comprehensive, the research team did not miss any search terms or relevant articles, and to confirm the quality of searches” (Sethi et al., 2021, p. 78). We searched the following 13 academic databases: Web of Science, Social Services Abstracts, Family and Society’s Studies Worldwide, Social Work Abstracts, Sociological Abstracts, Social Sciences Abstracts (H.W. Wilson), CINAHL, SCOPUS, CPI.Q., Gender Studies Database, Humanities International Complete, ProQuest Sociology Collection, and ProQuest Education Database. See Appendix 1 for a list of the databases and the number of hits in each database. These databases produced **156** articles. RA reviewed the list of 156 articles for duplicates. After removing the duplicates, **119** articles remained. RA and A1 scanned through the 119 articles. Only **33** papers were selected for the final screening.

### Phase Two: Final Article Selection

The RA downloaded and transferred these 33 articles into the Mendeley reference management software. The research team (RA, A1 and A2) reviewed the full text of the 33 articles to ensure that they met the inclusion criteria. After the full-text screening, rating, and a collaborative discussion, only **8** articles that addressed the inclusion criteria were included in the study (See Table 2). The final selection, in step two, had **8** papers from the Summon search engine.

Based on steps one and two screening, the total number of papers selected from steps one and two was **16** articles (**8** from the Summon Search Tool and **8** from individual databases) (See Table 2).

**Fig. 1:**
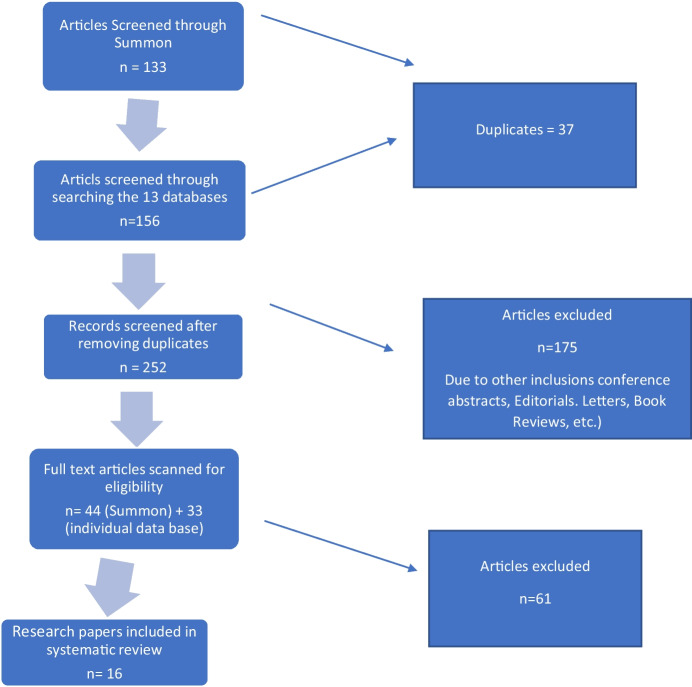
Research process for selecting the 16 scholarly research papers

**Fig. 2:**
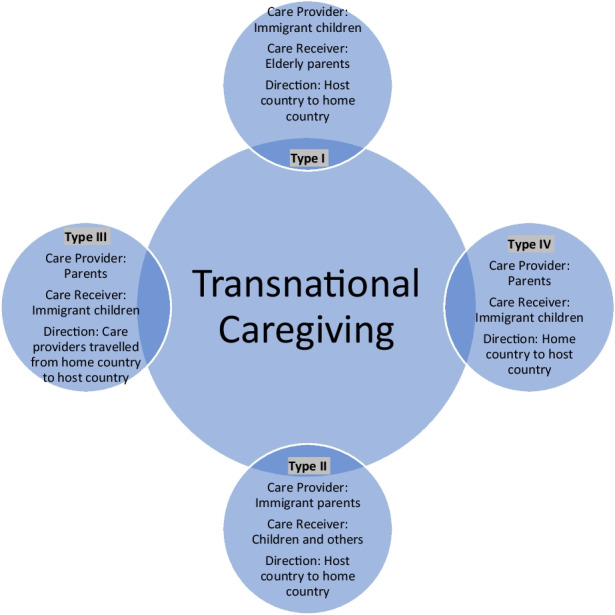
Typologies of transnational caregiving

Furthermore, in Table 3, we have detailed the characteristics of research papers on transnational caregiving in Canada. Similarly, Table 4 highlights the characteristics of research papers on transnational caregiving in the USA and Australia. Also, we illustrate the selection process of the final 16 research papers in Fig. 1. We have explained the typologies of transnational caregiving in Fig. 2. Appendix 1 outlines the number of journal articles selected by the individual database.

**Table 3:** Characteristics of research papers on transnational caregiving in Canada

S. No.	Author	Title	Target Ethnic Group	Caregiving Provider	Caregiving Receiver	Type and Main Research Area
1.	Bernhard et al. (2008)	Transnationalizing Families: Canadian Immigration Policy and the Spatial Fragmentation of Care-giving among Latin American Newcomers	Latin American women who experienced significant periods of parent-child separation because of migration	40 Latin American mothers residing in Toronto	Latin American children under 18 years of age residing in Colombia, Costa Rica, Guatemala, El Salvador, Ecuador, or Mexico	Type I This paper examines the causes and consequences of spatial dispersion for Latin American families living in Toronto.
2.	Gui and Koropeckyj-Cox (2016)	“I Am the Only Child of my Parents”: Perspectives on Future Elder Care for Parents among Chinese only-Children Living overseas	The only-child generation of China	20 young Chinese adults who were studying or working in Montreal, Canada	Parents of Chinese young adult migrant workers who were still living in China	Type II This paper explores the plans and concerns of the only-child generation of Chinese students and workers in Canada regarding their parents’ care and the challenges they expected to encounter in the future.
3.	Lewis (2001)	Universal Mother: Transnational Migration and the Human Rights of Black Women in the Americas	Afro-Caribbean women who migrate to North America as migrant workers	Afro-Caribbean women as migrant workers	Children of the Afro-Carribean migrant workers residing in home country	Type I This paper explores the human rights implications for Afro-Caribbean women who migrate to North America. Some of these migrant women take jobs as household workers and other caregivers (“domestics,“ home health aides, and nannies).

**Table 4:** Characteristics of research papers on transnational caregiving in USA and Australia

S. No.	Author	Title	Target Ethnic Group	Caregiving Provider	Caregiving Receiver	Type and Main Research Area
1	Ahmad (2016)	Transnational Caregiving for My Father: An Opportunity and a Blessing	Bangladeshi	A Bangladeshi immigrant daughter living in Australia	A critically-ill father living in Bangladesh	Type I This paper is based on personal experience of the author, a Bangladeshi woman, of this paper. She explained her stressful experience of providing long-distant care to her seriously ill father.
2	Amin and Ingman (2014)	Eldercare in the Transnational Setting: Insights from Bangladeshi Transnational Families in the United States	Bangladeshi immigrant men and women in the United States	21 Bangladeshi immigrant men and women living in the United States	Parents over 60 years old residing in Bangladesh	Type I This paper examines caregiving practices and outcomes of the transnational caregiving, focusing on understanding the caregivers’ stressors, mediators, and outcomes as described in Pearlin’s Stress Process Model (1990).
3	Baldassar (2015)	Guilty feelings and the guilt trip: Emotions and motivation in migration and transnational caregiving	Italian transnational families	Italian adult migrant children living in Australia	Parents living in Italy	Type I This paper explores the experience of ‘guilt’ as a motivating emotion in the migration process.
4	Brijnath (2009)	Familial Bonds and Boarding Passes: Understanding Caregiving in a Transnational Context	Indian migrants	An Indian granddaughter who lives in Australia	An Indian grandmother who resides in USA	Type I The purpose of this article is, by actualizing Yeates’ (2005) theory using a personal case study and drawing on Baldassar’s (2007a, b) work, to build more complex analyses of the relationships between the various caregivers in a global care chain.
5	Dhar (2011a, b)	Transnational Caregiving: Part 1, Caring for Family Relations Across Nations	General discussion paper	n.a.	n.a.	This paper concerns how globalization and the aging of the world’s population are affecting the already complex issue of intergenerational transnational caregiving.
6	Dhar (2011a, b)	Transnational Caregiving: Part 2, Caring for Family Relations Across Nations	General discussion paper	n.a.	n.a.	This paper concerns how globalization and the aging of the world’s population are affecting the already complex issue of intergenerational transnational caregiving.
7	Heymann et al. (2009)	The impact of migration on the well-being of transnational families: new data from sending communities in Mexico	Mexican children in migrant households	Mexican adult immigrants in USA	Mexican households	Type II This paper assesses the overall impact of migration on the well-being of the families involved including economic, health, education, and child development.
8	Lahaie et al. (2009)	Work and family divided across borders: the impact of parental migration on Mexican children in transnational families	Mexican children in migrant households	Adult member of the Mexican family who migrated to USA	Mexican children left behind in Mexico	Type II This paper explores the impact of immigration on children who experience separation from the parents and left behind in changing family environments.
9	Lee et al. (2015)	Caring from Afar: Asian H1B Migrant Workers and Aging Parents	Chinese/Taiwanese, Indian and Korean H1B workers residing in the SF Bay Area	21 Chinese/ Taiwanese, Korean, and Indian H1B workers	Aging parents over 60 years old in-home countries	Type I This paper explores the challenges that H1B Asian immigrants face when providing care from a distance and discusses the types of Care that H1B Asian immigrants provide for an aging parent abroad.
10	Şenyürekli and Detzner (2008)	Intergenerational Relationships in a Transnational Context: The Case of Turkish Families	Turkish	28 Turkish immigrants living in USA	Parents in Turkey	Type I This paper explores how intergenerational relationships are experienced in a transnational context; the nature of these connections; and how they help immigrants stay close to family members back home.
11	Singh et al. (2010)	Remittances as a Currency of Care: A Focus on Twice Migrants among the Indian Diaspora in Australia	Indians and other multiple migrants	19 migrants identifying with the Indian diaspora in Australia. Multiple migrations from Malaysia, Singapore, Kenya and the UK and onward migration from Australia to Singapore, the UK, Canada, and the USA.	Different family members of immigrants in home country	Type I This paper focuses on the ways in which family remittances contribute to a migrant’s sense of belonging to his or her transnational family as well as the tensions in caring and providing remittances.
12	Tung (2000)	The Cost of Caring: The Social Reproductive Labor of Filipina Live-in Home Health Caregivers	Filipina Community Workers	Filipina Community Workers in Southern California	Children of the Filipina Workers residing in their home country	Type II This paper examines the social reproductive labour that shapes transnational families’ lives and the cost of caring and mothering from afar.
13	Wilding and Baldassar (2018)	Ageing, migration and new media: The significance of transnational care	General discussion paper	Transnational families	Ageing parents	Type I This paper discusses the care network of older people and global, transnational, and virtual contexts within ageing and aged care.

It is important to note that, due to the lack of literature **exclusively** focused on TCEs, we included papers in which TCEs were not the focus. Nevertheless, these papers helped us address our research questions and advance our knowledge about TCEs experiences. For example, Ahmad (2016) discussed her experiences as a transnational caregiver to her father. Amin and Ingman (2014) focused on the emotional impact of caregiving across international borders. From the 16 selected papers, only the following authors exclusively addressed the TCE’s experiences: Canada = 1 (Lewis, 2001); USA = 4 (Tung, 2000; Heymann et al., 2009; Lahaie et al. 2009; Lee et al., 2015); and Australia = 0.

## Results

We have divided the findings into descriptive findings (relates to the characteristics of transnational caregiving) and analytical findings (provide an in-depth analysis of the 16 selected studies).

### Descriptive Findings

This review aims to provide an overview of TCEs’ experiences in the USA and Australia. Of the 16 selected articles, researchers used qualitative methods in ten papers, quantitative methods in two papers, and an exploratory approach in four articles. Specific methodologies were identified in eight of the ten qualitative studies: three studies used a combination of an interview and questionnaire; four studies used in-depth semi-structured interviews, and one study used to interview and participant observation. The others did not describe a specific methodology but described various qualitative methods or techniques. Participants in the selected studies were recruited by multiple methods, including multi-site quota sampling or snowball sampling. The 16 selected studies involved diverse ethnic groups, including Latin American, Chinese, Afro-Caribbean, Bangladeshi, Indian, Mexican, Turkish, Filipina, Italian, Taiwanese, and Korean. Seven studies included male and female participants, and three studies only included female participants. Grounded theory was the qualitative approach used in four studies to understand the relationship between the data and theory. One study used a thematic analysis approach to analyze data. The two quantitative studies used multivariate logistic regression models to address their research questions.

#### Transnational Caregiving in the Canadian Context

The detailed search and literature review on TCEs’ experiences in Canada affirms that very limited research has been undertaken on this topic. We located only three papers on Canadian immigrants’ experiences of TCEs. Table 3 provides a summary of the selected papers. The first paper in Table 3 focuses on Latin American mothers’ practices of providing care to their children in their home countries and their excruciating experience of mothering from afar. The second paper focuses on Chinese immigrant workers and students who belong to the one-child policy generation of China. The author explores their perceptions of the traditional Chinese value of familial piety and their concerns about providing care to their parents from afar. The last paper presents the legal case of *Baker v. Canada* and discusses the deportation of a Jamaican mother with children in both Canada and Jamaica.

#### Transnational Caregiving in the non-Canadian Context (the USA and Australian Context)

Because of the paucity of literature written on TCEs in Canada, we broadened the scope of our search to transnational caregiving in the United States of America and Australia. This section provides a descriptive summary of the thirteen (n = 13) research papers that fulfilled the inclusion criteria. These papers were very useful in building our understanding and knowledge of the phenomenon of transnational caregiving and its consequence on transnational families. These papers affirm that transnational caregiving is a sensitive and vital issue that has not been extensively explored and studied.

### Analytic Findings

We used content and thematic analysis to synthesize the selected papers. Content analysis is a systematic, replicable technique for constructing content categories from the text (Neuendorf, 2016). We adhered to thematic analysis to identify the generated analytical themes (Morse, 1991; Morse & Field, 1995). The RA extracted the codes or emerging patterns from the final selected papers. The research team met to review/discuss the codes and reach a consensus on the final codes. The RA identified and compared code frequencies in the final synthesis and synthesized the data into final themes (Morse, 1991). The research team met again and agreed upon the final themes.

In total, we identified sixteen codes. We grouped these codes into four categories/themes concerning their relevance with the objectives or/and research questions, in order of significance: (1) Typologies of Transnational Caregiving; (2) Multiple Forms of Transnational Caregiving; (3) Challenges and Dilemmas of Transnational Caregiving; and (4) Influence/Role of Cultural, Historical, and Religious/Spiritual Elements on Notion and Practices of Transnational Caregiving.

We will be focusing on the first three most significant themes: (1) Typologies of Transnational Caregiving; (2) Multiple Forms of Transnational Caregiving; and (3) Challenges and dilemmas of Transnational Caregiving. The detailed discussion of the theme (4) “Influence/Role of Cultural, Historical, and Religious/Spiritual Elements on Notion and Practices of Transnational Caregiving” is beyond the scope of this paper.

#### Typologies of Transnational Caregiving

The content analysis of selected papers (Neuendorf, 2016) in this review highlights that transnational caregiving is not a multidirectional phenomenon; it can be divided into four types of transnational caregiving.

These involve: (1) immigrant children providing care from the host country to their parents in the home country and/or immigrant family member (e.g., women) providing care to parent/spouse/children in the host country; (2) immigrant parents providing care from the host country to their children and other family members in the home country; (3) parents travelling from the home country to provide care to their children and grandchildren in the host country; and (4) parents in the home country providing distant-care to their immigrated children in the host country.

From the 16 selected papers, nine papers discussed transnational caregiving in the context of caregiving experiences of immigrants residing in Canada, the USA, and Australia to their elderly parents in the home country of concern (Type I), and five papers explained the experiences and impact of transnational parenting from a host country to their children in the home country (Type II). Type III and Type IV of transnational caregiving do not fit the study inclusion criteria of providing care from a host country to a home country.

Our previous studies confirm other literature that despite racialized newcomers’ skills and education, they struggle in the labour market as the education, knowledge, and experience obtained in their country of origin are not recognized in Canada (Sethi, 2016; Sethi & Williams, 2016; Sethi & Williams, 2017). As a result, their household income is typically less than that of a native-born (Guo, 2013). Often, they need financial support that parents or other family members like siblings provide to them from their home country. In our view, these contributions, particularly parents make in helping immigrants resettle in Canada, are rarely acknowledged. Neysmith and Zhou (2013) recognized in their paper the care that non-immigrant parents provide to their children in the host country is unpaid transnational care. Neysmith and Zhou (2013) shared the experiences of thirty-six Chinese grandparents in providing transnational caregiving to their grandchildren in Canada, concluding that:

International migration, as ‘a life-long process of complex interaction between individuals and groups who often live far apart’ (Lunt, 2009: 244), has presented extensive impacts on families transcending national borders. Seniors’ continuing contributions to their adult children’s family economy, and indirect contribution to the latter’s participation in the labour market and integration into the host society through transnational caregiving, also suggest a new pattern of resource redistribution - not resources exchange, given its one-directional nature - across generations and countries (p. 293).

#### Multiple Forms of Transnational Caregiving

The nature of “care” in the typologies mentioned above varies and can be grouped into three primary practices of transnational care: financial care, emotional care, and practical care.

- *Financial care* – sending money in the form of remittances and gifts is the most common form of transnational care that is cited in half of the selected papers. It is noted that the pattern of financial care from immigrant families to their home country is usually provided routinely. The impact of remittances in selected papers is discussed in various ways: from providing for care-recipients medical care, hiring a medical professional to take care of their parents, fulfilling a cultural obligation, and/or contributing to the national economy of the home country. For some, “financial remittance is a parent’s primary source of income.” (Lee et al., 2015, p. 325).

Singh et al.’s (2010) paper offers two key insights regarding remittances as a form of transnational care that have not been widely discussed in the literature. These authors explored the experiences of the Indian diaspora in Australia. First, they found that conflict arises within migrant families when “relatives in the home country perceive the quantity of money in remittances, is lacking when weighed against the physical caregiving that the migrants cannot routinely provide” (Singh et al., 2010, p. 255). This is the center of tension for transnational families. Second, the common perception that remittances flow only from the North to the South is inaccurate. Remittances in the form of cash and kinds of flow from the South to the North. Parents of immigrant families living in their home countries support their children settling down in the new country by providing care of all types, including money. Bernhard et al. (2008) report that participants in their research study stated they had “received money from their non-immigrant parents and siblings” (p. 17).

- *Practical care* – entails performing instrumental activities (meal preparation, bathing, dressing, and shopping) by paying actual visits; and planning for in-home care for children and/or oversight of medical care for elderly parents by involving siblings and formal long-term care institutions. Practical care is the only form of transnational care that cannot be provided on a regular basis by both parties. However, transnational caring networks emerged as an interesting concept in this literature review. Lee et al.’s (2015) research with highly skilled Asian transnational caregivers working in the USA revealed that immigrants developed caring networks that included, in most cases, siblings, relatives, or family friends who live close to their parents to provide care to ageing parents/grandparents (Lee et al., 2015). In some cases, professional healthcare workers were encompassed in these care networks (Ahmad, 2016). An interesting point here is that, in most cases, the immigrant family members who live far away still provide practical care by playing an instrumental role in forming these caring networks in Canada, Australia (Brijnath, 2009, Dhar, 2011a, b, Baldasssar, 2011, Ahmad, 2016), and the United States of America (Amin & Ingman, 2014). Nevertheless, Amin and Ingman (2014, p. 315) observed that even though the Bangladeshi immigrants living in the USA arranged for transnational care, there was a sense “of loss of control over the caregiving process”, as they were not physically present to provide the hands-on care to their elderly parents in Bangladesh.
- *Emotional care* - involves giving emotional support through regular telephone calls and/or communicating through other social media such as WhatsApp, Zoom, or Skype. Immigrant children in selected papers reported that they regularly made calls to their parents using Skype and other forms of communication technology. Simultaneously, they stated that parents and siblings also make calls to their family members in the host country and provide emotional care that they need to settle down in a new country.

From a gender perspective, while both male and female transnational migrants felt a cultural and familial obligation to provide care, Amin and Ingman (2014) note that female transnational caregivers in the US spent more time emotionally supporting their elderly family members. The female caregivers’ conversations with their parents or other family members were more intimate and lengthier than the male caregivers. Similarly, female TCEs were more likely than men to take time off from work to travel home to provide personal care to their elderly parents (Bernhard et al., 2008; Lee et al., 2015).

The review reveals that the frequency of care depends on the form/practice of the care and the person(s) who receives the care. For example, if the care receiver is a parent or child of the caregiver (i.e., Type I and Type II transnational caregiving) then the financial and emotional care were given on regular basis, even in some cases on daily or weekly grounds, for an extended period of times; but, the physical care, due to the vast physical proximity, is given on casual basis for a short time.

#### Challenges and Dilemmas of Transnational Caregiving

Transnational families providing caregiving to their loved ones experience many structural and psychological challenges. In general, when a family member migrates from a developing to an industrialized nation, that occasion is celebrated at the family level and regarded as an achievement in their society. However, at the time of immigration, caregiving is not thought through thoroughly in most cases, and it is taken for granted by family members.

Most papers explained the participants’ perspectives and experiences of their dilemmas from being far away from their families. Providing care and obliging cultural/family obligations from a distance is one of those challenges. Some TCEs developed formal (a workplace soccer club) or informal networks (friendship care networks) to support each other (Lee et al., 2015). These care networks included siblings, relatives, or family friends who lived near their ageing parents/relatives. In some cases, professional healthcare workers were also included in these care networks. The interesting point here is that, in most cases, the immigrant family members who live far away play a significant instrumental role in forming these caring networks.

An analysis of Brijnath’s (2009) research demonstrates that “the constant negotiations, communications, and reciprocities that characterize these relationships illustrate how care work in a transnational context is imbued with emotional labour” (p. 98). It is essential to differentiate between the concept of *emotional labour* in the context of international global care chains (Hochschild, 2000) and *emotional investment* of TCEs. Global care chains were first used by Arlie Hochschild (2000) in the context of the mass migration of labourers (largely Filipino female domestic workers) to the global North as nannies or maids. As a result of globalization of the labour market and demand for domestic workers, these women leave their children behind to provide emotional labour to other’s children in a rich country, thus depriving their children of motherly care. However, TCEs are typically middle-class and higher-income migrants (Amin & Ingman, 2014) who invest in intensive emotional labour beyond motherly care to fulfill their financial, practical, and emotional care obligations to family members in their country of origin. Such emotional labour in the transnational context can lead to difficult/troubled emotions. For example, feelings of guilt, fear, shame, sacrifice, worries, anxiety, stress, burnout, hopelessness, and disempowerment were highlighted in all the papers. Amin and Ingman (2014) observed that their guilt and chronic anxiety could lead to negative physical and mental health outcomes for transnational families, including depression. What is important to note is that such transnational caregiving is not static; it fluctuates and is mediated “by migration stages, historical contexts (including available technologies), family life-cycles and histories; but also, like the bonds of kinship which define them, of their persistence over time and distance” (Baldassar, 2007a: p. 294).

The concept of “guilt” is the emotion that most appeared during the content analysis of papers selected for this review, regardless of the country of origin and the country of resettlement. In 1994, Baumeister et al. (1994, p. 243) challenged the intrapsychic concept of guilt by proposing that guilt is “something that happens between people rather than inside them.” They further argued that guilt arises when an individual feels emotionally distressed about their “actions, inaction, circumstances, or intentions” (Baumeister et al., 1994, p. 245). Baldassar’s (2015) work on transnational eldercare in Australia builds on the Baumeister et al. (1994) relational view of guilt. She notes that migrants experienced guilt due to their inability to effectively fulfill their moral obligations towards their ageing parents due to the vast physical distance. Similarly, our systematic review of literature has also shown that in transnational settings, TCEs experienced guilt as they were unable to be physically present to provide practical (e.g., taking parents to the doctor) or personal care to their parents due to the challenges of geographical distance (Amin & Ingman, 2014). Bernhard et al. (2008) discuss the experience of a Colombian newcomer to Toronto who was separated from her children for two years:

The family nucleus was the most important thing for me. The most important thing in the world. And to all of a sudden come to be dismantled like this, from one day to the next it is all over and we are separated….It is hard…. Sometimes I feel guilty and ask myself if it was all worth it, if it was really worth it to come here leaving three children behind, or is it that only money is important? Or is it only important to be here? And what about human values and respect? What is the place of family then? These are questions that I ask myself all the time. (pp. 19–20)

Similarly, Ahmad (2016) shares the guilt and anguish of not being able to be physically available to her ill and aging father after her migration to Australia:

Caught between being a daughter and a wife and mother, I have struggled for many years with the anguish and guilt that has followed me like a shadow ever since family circumstances forced me to leave my father to the care of my other siblings and formal carers in Bangladesh and join my husband who lived abroad. How I wanted to continue to take care of my father! But alas! Life seemed to move in another direction for me. .. I was often overwhelmed with the guilt and competing priorities associated with caring for a father who needed me and a husband and a young daughter who wanted me. (p. 6).

Amin & Ingman’s (2014) found that transnational caregivers who had a family, friend, or extended relative who could help their parents experienced less anxiety and guilt compared to those whose parents were isolated and alone. However, Brijnath’s (2009) study result suggests that there is a sense of relief that being so far away, one does “not have to endure the physical care burdens that proximity brings” (p. 92). Nevertheless, this sense of relief is overlaid with shame and guilt for being 16,000 km away and not doing enough and being “so useless” (Brijnath, 2009, p. 92). Like Baldassar’s (2015) observation, the analytical findings of this literature review demonstrate that guilt can be a decisive motivating factor for adult children to provide transnational care to ageing parents in their home county, both emotionally and financially. But this experience “incurs psychological, emotional, and financial costs” (Lee et al., 2015, p. 328). This is the dilemma that transnational families face. On the one hand, they are considered successful and feel satisfied in fulfilling their family responsibilities and cultural obligations. On the other hand, there are psychological, emotional, and financial problems resulting from transnational care. For example, while TCEs considered it an obligation and privilege to reciprocate the care that their parents had provided over the years through financial remittance, sending money home when TCEs had limited income created financial hardships and uncertainties (Lee et al., 2015; Singh et al., 2010; Kodwo-Nyameazea & Nguyen, 2008). Even though TCEs in all the papers had the desire to visit their family members, financial resources and longer work leave are needed in order for TCEs to make frequent visits to their country of origin (Şenyürekli & Detzner, 2008). Others (Gui & Koropeckyj-Cox, 2016) observe that, based on the Chinese tradition of filial piety, family members are expected to take care of their elderly parents and relatives. However, adult children are often forced to put their parents and grandparents in long-term care institutions as they are unable to provide care due to busy work schedules.

Still, some positive outcomes of transnational caregiving are found in this literature review. For example, a few selected papers reported that they found the whole experience of providing care to their adult parents very rewarding. The “performance of Indian feminity” is displaced by South-Asian women in Brijnath’s (2009, p. 93) research. These women fulfilled their commitment by regularly staying in touch and spending their vacations providing practical care to their elderly parents in their native country. Such performativity helped ease the complex feelings of guilt as they had lived to the ideals of a “good” Indian woman (Brijnath, 2009, p. 93).

## Discussion

A key finding of this review is ‘the essence’ of transnational caregiving: that diasporas in the USA, Australia, and Canada want to maintain their ties with their home countries. Transnational families strive to maintain and sustain relations with the home country by communicating with their parents, children, siblings, and extended relatives. They also provide them with practical, financial, and emotional care that includes remittances and gifts; however, the pattern of transnational caregiving differs depending upon the type of care. TCEs travel home to provide personal or hands-on care to their elderly family member (Lee et al., 2015). Dhar (2011a) explained, “The concept of transnationalism is not new. What may be considered new is the ‘term’ that is used to discuss the relationships that immigrants sustain between the home and the host country” (p. 62). In this context, Neysmith and Zhou (2013) define transnationalism as a “process ‘by which immigrants forge and sustain multi-stranded social relations that link together their societies of origin and settlement’” (Basch, Schiller, & Blanc, as cited in p. 146).

Our systematic review of the literature affirms that caregiving across geographies is a complex and multi-layered process. Transnational caregiving can be both fulfilling and stressful. For example, Ahmad (2016, p. 6) considers caregiving as “an opportunity and a blessing”, and some immigrants feel privileged to be able to fulfill their cultural, moral, and religious obligations (Amin & Ingman, 2014). However, when TCEs cannot be physically present to take care of their loved ones, they experience guilt, anxiety, and helplessness (Brijnath, 2009; Baldassar, 2011, 2015, 2016; Ahmad, 2016; Amin & Ingman, 2014). There is a sense of loss at being separated by distance (Baldassar, 2008; ). There is evidence to suggest that TCEs often deal with this guilt by giving financial and practical support (e.g., ensuring that the parents have formal or informal supports) to their families in the country of origin (Bernhard et al., 2008; Lee et al., 2015; Miyawwaki & Hooyman, 2021; Amin & Hungman, 2014). However, given limited income, especially for new immigrants, sending money can create a financial burden for themselves and their families in the country of resettlement (Lee et al., 2015; Sethi 2014; Sethi & Williams, 2017; Sethi, 2022). It is interesting to note that Amin and Ingman (2014) observe that providing remittance is not a strong risk factor for caregiver burden.

A critical gap that became evident in conducting this work presented herein is the paucity of research exclusively focused on TCEs experiences. Even the papers that explored TCEs experiences were focused on specific workers. For example, Tung (2000) explored the experiences of Filipino home healthcare workers from a social reproductive labour perspective. Lee et al. (2015) studied the experiences of Asian migrant workers in the US in the H-1B program (temporary employment of workers on a non-immigrant basis in specialty occupations). Heymann et al. (2009) and Lahaie et al. (2009) used large-scale secondary data analysis from a Transnational/Global Working Families survey to capture the impact of parental migration on children/families left behind. Given the global aging population, future research must examine the experience of a diverse sample of TCEs at the intersection of gender, geography (where the individual lives and the country of migration) and immigration status (e.g., permanent resident, citizen, international student, or foreign worker), given that immigration status is a social determinant of health (Castañeda et al., 2015). While some scholars have explored the interplay between caregiving and employment (see Fast et al., 2013), country-specific studies are needed to compare and contrast the impact of different types of employment opportunities, employment and immigration policies, and degree of gender parity on transnational caregiving. This is particularly important as countries compete for skilled labour given the shortages that exist. For example, research supports the fact that immigrant and racialized women are largely employed in precarious healthcare jobs (Sethi, 2020). Further, workplace characteristics (such as business size and the type of sector) determine if employers are supportive of CFWPs (Ireson et al., 2018), with large businesses with Human Resource (HR) departments more likely to offer CFWPs when compared to businesses without a HR department (Vuksan et al., 2012a, b). Sectors that are known to have deep pockets, such as the financial, technology, pharmaceutical and public/government, are more likely to offer CFWPs given their interest in recruiting and retaining highly skilled employees. Given the presence or absence of gender-dominance across sectors (e.g., female dominance in the service sector and male dominance in construction and extraction sectors), and the important role that both sex and gender play in caregiving expectations, CFWPs are more likely to be needed and utilized in female-dominated sectors. Given that women represent a larger proportion of unskilled jobs characterized by lower-pay and benefits, they are particularly vulnerable to being caught at the intersection of work and care, regardless of the nature of their employment (Vuksan et al., 2012a, b).

Further, local, national, and transnational policies (such as health care, and elderly care) impact TCE’s experiences of providing care to their elderly family members in the country of origin and resettlement (Castañeda et al., 2015; Sethi, 2022). Miyawaki and Hooyman (2021, p. 8) make a poignant observation that “the existence of national and employer policies, such as flexible family leave, and the ease of access to technology, are part of the political context affecting transnational care. In most instances, inflexible family leave policies or immigrants being ineligible for such leaves make extended visits home difficult.” Given their very limited availability, there is sufficient evidence to suggest the need for CFWPs, such as shared work, flex work, compressed work weeks, non-contiguous leaves, and a wide range of other accommodations, to support carer-employees, broadly defined, in the workplace (Lorenz et al., 2021; Ireson et al., 2018). Lack of workplace support via CFWPs leads to consequences such as: carer-employees leaving the workforce/missing work; premature retirement; reduced productivity; health problems; and increased costs to employers (Peters & Wilson, 2017). The uptake of CFWPs would require a significant workplace culture change that would recognize and accommodate unpaid elder/adult care work in a similar way as unpaid care work of infants and children. When discussing TCEs specifically, their requirements need to be accommodated in specific ways, given the unique characteristics of transnational caring. For example, the various investments in international travel to one’s home country require flexibility in the provision of CFWPs for TCEs. Such flexibility requires a case-by-case approach that would allow tailored solutions, providing TCEs to not only take care of their families in the resettlement country, but also care for those family living in their home country while, simultaneously remaining engaged in essential paid work roles (Amin & Ingman, 2014; Sethi & Williams, 2021).

While our study was focused on TCEs (primarily an adult child) providing care to an adult family member (an aging parent, relative, or friend), the findings challenge the notion that international caregiving practices and experiences are uni-directional in which only adult children provide care to their ageing parents/grandparents in the home country. Neysmith and Zhou (2013, p. 145) report that many older adults (parents and grandparents) support their children financially and through “caring labour” so that those children can contribute to the Canadian economy. However, this is “at a cost to their own quality of life, costs that are amplified by Canadian visa requirements that were designed to control the supply of immigrant labour as well as the flow of ‘nonproductive’ migrants” (Neysmith & Zhou, 2013, p. 145). This finding challenges the notion that international caregiving practices and experiences are uni-directional in which only adult children provide care to their ageing parents/grandparents in the home country. Thus, a “culturally complex” awareness of family care requires a transnational lens that integrates the dimensions of transnational caregiving simultaneously from both sides (the host country and home country) (Andruske & O’Connor, 2020, p. 54). Yet, we know very little about the financial contribution and emotional labour of parents and grandparents in the resettlement of immigrant children. Further research is needed to understand the experiences and unpaid caregiving of older immigrant adults who enter Canada as visitors and provide practical, financial, and emotional care to their children and grandchildren (Zhou, 2013). Such reciprocal nature of transnational caregiving should be studied from a new angle.

The assumption that immigrants are economically well-off in the host country and are the sole provider of transnational care to their home country must be challenged. Parents’ contributions to their children must be recognized and supported (Neysmith & Zhou, 2013). For instance, *Canada’s Parent and Grandparent Super Visa* are promoted as encouraging family reunification. However, the financial requirements and investment from adult children as sponsors penalize low-income households (Spitzer et al., 2006). Ferrer has argued that the streamlined super visa program “categorize older adults as ‘visitors’ who must be surveilled and made ineligible for state benefits because of common perceptions that they consume valuable and scarce resources” (2015, p. 264). Even if parents are granted a visa to Canada, they are expected to buy health insurance. If we take into serious consideration the role they are playing as caregivers for their children and/or grandchildren living in Canada, it seems they are being penalized for providing such care.

Our results affirm the increasingly important role that virtual communications play in facilitating migrant networks and strengthening family ties across international borders (Dekker & Engbersen, 2014). International travel restrictions and border closures disrupted transnational care as “travel is a necessary part of fulfilling familial obligations and maintaining a sense of ‘familyhood’ and belonging across borders” (Ferazzoli & Walsh, 2021, para 2). Nonetheless, one of the potential positive aspects of pandemic travel restrictions may be the increase in TCEs’ emotional and affective components due to frequent communications. Baldassar has argued that in the absence of physical closeness, international families create a virtual “shared (co)presence” through telephone, text, and other forms of technology (2008, p. 247) through which they perform emotional labour. Madianou (2016) proposes that due to the availability and affordability of several media platforms, transnational families experience and intentionally manage their relationships by choosing a specific type of communication medium for particular purposes. “Polymedia environments” facilitate various kinds of presences: “connected presence,” (e.g., synchronous platforms such as WhatsApp); “co-presence by proxy” (e.g., visual content such as photos, animated visuals, infographics, etc. on social media) and “ambient co-presence” (e.g., newsfeeds that can include an update on photos, videos, links to websites and stories, etc.) (Madianou, 2016, p. 187). The availability of accessible and reliable technology and new social media platforms will undoubtedly have a long-lasting impact on transnational engagement and familial care. Despite the integration of new social media platforms in our daily lives, “longing, missing and nostalgia are best resolved through physical co-presence; actually, being bodily present with the longed-for person or in the longed-for place so as to experience them fully, with all five senses” (Baldassar, 2008, p. 252). We suggest that future studies must consider the consequences of the COVID-19 pandemic on transnational family relations. For instance, in Canada and other OECD countries, due to job losses and the overrepresentation of immigrants in precarious employment, the pandemic has negatively affected the financial and health of ethnic minorities much more than the native-born (Mohsin, 2022).

## Conclusion and Study Implications

Our systematic review of the literature on TCEs affirms and signifies the issues related to transnational caregiving, ageing, and migration. The literature shows that international migrants maintain strong ties to their home country while simultaneously engaging in allegiances in a host country (Levitt, 2019). The participation of activities in the resettlement country can potentially transform the country’s economy, culture, and political movements (Levitt, 2019). “When migrants live their lives across national borders, they challenge many long-held assumptions about membership, development, and equity” (Levitt, 2019, para. 16). Understanding this reality in this globalized and interconnected world requires timely and innovative policy responses (Levitt, 2019). Current policy models in care work consider caregiving within national borders. Policymakers must integrate the resettlement experiences of transnational caregivers within their country of origin and country of resettlement.

COVID-19 highlighted the health care crisis and inequity in caregiving resources within Canada (Das Gupta, 2020). However, there was no discussion on the unique challenges of adult children providing care to ageing parents across thousands of miles during the pandemic. For example, as a transnational caregiver, the first author could not travel home due to travel restrictions imposed by the pandemic or partake in a family member’s cultural death ritual. While Baldassar and colleagues’ groundbreaking work on transnational families from the perspectives of adult children to their ageing parents has brought to light the complexities of transnational caregiving, we know very little about the caregiving experiences of TCEs. More research is needed to understand the effect of transnational caregiving on migrants’ economic, social, and political integration. A growing number of racialized newcomers, especially women, are employed in low and precarious health care jobs (Zagrodney & Saks, 2017).

The new Canadian immigration policies (such as parent and grandparent super visa) are institutionalizing the geographic separation of family members in immigration entrance categories that used to accommodate the immigration of intact (nuclear) families. (Bernhard et al., 2008, p. 22). In light of this policy shift and aging demographics, it is not erroneous to assume that parents/grandparents can face barriers to family reunification. Although our article is about TCEs, the moral and ethical obligation of adult children to take care of their parents left behind has broader implications for informing workplace policies. Employers must be engaged in the conversations around the financial, emotional, and time investment required to maintain transnational communication. Given the growing care needs of an ageing population globally, supporting migrants to balance their work duties and fulfill caregiving obligations across national borders is economically productive to the employer and employee. Studies have shown that caregiver-friendly workplace policies and programs benefit the employer due to outcomes, such as less employee turnover, retention of skilled employees, and reduced absenteeism (Ireson et al., 2018; Lorenz et al., 2021). A recent study found that supporting carer-employees through such workplace policies sustains their dual roles as paid employees and unpaid caregivers (Wang et al., 2018).

Our analysis confirms the multidirectional flow of transnational care. We are aware that TCEs are not a homogenous group. Furthermore, in the last decade, there has been a strong growth of international students in North America and Australia. As international students attain permanent immigration and become part of the labour force, they will impact these countries’ economies in years to come (El-Assal, 2020; Crossman et al., 2021). It can be expected that they will provide financial and other transnational care to their ageing parents/relatives left behind in the source country. Policymakers must use a transnational and intersectional lens in policymaking processes. This entails paying attention to the multi-faceted migration realities that account for intersections of migrants’ social locations (age, race, socioeconomic class, ethnicity, and gender).

Another critical issue is the exponential growth of media platforms. Their dynamic nature requires unpacking the nuances of media in mediating and navigating transnational relationships (Madianou, 2016). Most of us have used Zoom, Facetime, Google Hangouts, and other media platforms for work and social connections; transnational communities relied exclusively on digital technologies for international communication. Many of the healthcare resources (such as counselling) were delivered virtually. We cannot assume that older adults and/or newcomers have the literacy and adequate communication infrastructure for international communications. There is concern that the lack of transnational connections can have a detrimental health impact on older adults and their children (Wilding & Baldssar, 2018). There is an urgency for the government to ensure that newcomers have the literacy and formal and informal resources necessary for transnational care.

## Data Availability

Data sharing not applicable to this article as no datasets were generated or analysed during the current study.

## Appendix 1 - Number of Journal Articles by Individual Database

**Table:**

S. No.	Database	Search Terms	Total Results	New Articles	Selected Articles
1	Family and Society Studies Worldwide 2000–2017	(Transnational Caregiving employees) OR (Transnational Care giving employees) OR (Transnational Care-giving employees) and Canada	17	15	3
2	Social Work Abstracts 2013–2013	Transnational Caregiving Employees	1	1	0
3	SCOPUS 2006–2017	(“Transnational caregiving”) OR (“Transnational care giving”) OR (“Transnational care-giving”) AND “Canada”	4	4	2
4	CPI.Q 2004–2016	Transnational Caregiving Employees	9	5	0
5	Social Sciences Abstracts (H.W. Wilson) 1998–2017	Transnational Caregiving Employees AND Canada	44	27	0
6	CINAHL 2008–2016	Transnational Caregiving Employees	6	4	1
7	Gender Studies Database 1989–2017	Transnational Caregiving Employees	36	32	0
8	Humanities International Complete 2005–2017	Transnational Caregiving Employees	18	18	0
9	ProQuest Sociology Collection				
	1998–2017	(“Transnational caregiving”) AND “Canada”	21	13	2
	Total		**156**	**119**	**8**

## Footnotes

1 A systematic review differs from traditional reviews in that it uses a replicable and transparent approach designed to minimize bias (Benach et al., 2010, p. 210).

2 Available for free download in either English or French at https://www.csagroup.org/store/search-results/?search=all~~carer-inclusive%20and%20accommodating%20organizations%20standard]

## References

[CR1] Ahmad M (2016). Transnational caregiving for my father: an opportunity and a blessing. Journal of Social Work in End-of-Life & Palliative Care.

[CR2] Amin I, Ingman S (2014). Eldercare in the transnational setting: insights from bangladeshi transnational families in the United States. Journal of Cross-Cultural Gerontology.

[CR3] Andruske CL, O’Connor D (2020). Family care across diverse cultures: re-envisioning using a transnational lens. Journal of Aging Studies.

[CR4] Arksey, H. & O'Malley, L. (2005) Scoping studies: towards a methodological framework, International Journal of Social Research Methodology, 8(1), 19–32, 10.1080/136455703200011961

[CR5] Baldassar L (2007). Transnational families and the provision of moral and emotional support: the relationship between truth and distance. Identities.

[CR6] Baldassar L (2007). Transnational families and aged care: the mobility of care and the migrancy of ageing. Journal of Ethnic and Migration Studies.

[CR7] Baldassar L (2008). Missing kin and longing to be together: emotions and the construction of co-presence in transnational relationships. Journal of Intercultural Studies.

[CR8] Baldassar L (2011). Italian migrants in Australia and their relationship to Italy: return visits, transnational caregiving and the second generation. Journal of Mediterranean Studies.

[CR9] Baldassar, L. (2013). Locating transnational care circulation in migration and family studies. *Transnational Families Migration and the Circulation of Care*, 41–74. 10.4324/9780203077535-8

[CR10] Baldassar L (2015). Guilty feelings and the guilt trip: emotions and motivation in migration and transnational caregiving. Emotion Space and Society.

[CR11] Baldassar L (2016). De-demonizing distance in mobile family lives: co‐presence, care circulation and polymedia as vibrant matter. Global Networks.

[CR12] Baldassar, L., Baldock, C. V., & Wilding, R. (2007). *Families caring across borders: migration, ageing, and transnational caregiving*. Palgrave Macmillan.

[CR13] Baldassar L, Nedelcu M, Merla L, Wilding R (2016). ICT-based co-presence in transnational families and communities: challenging the premise of face-to-face proximity in sustaining relationships. Global Networks.

[CR14] Baldassar L, Pyke J, Ben-Moshe D (2017). The vietnamese in Australia: Diaspora identity, intra-group tensions, transnational ties and “victim” status. Journal of Ethnic and Migration Studies.

[CR15] Benach J, Castedo A, Solar O, Martínez JM, Vergara M, Amable M, Buxó M, Demiral Y, Muntaner C (2010). Methods for the study of Employment Relations and Health Inequalities in a global context. International Journal of Health Services.

[CR16] Bernhard, J. K., Landolt, P., & Goldring, L. (2008). Transnationalizing families: canadian immigration policy and the spatial fragmentation of care-giving among latin american newcomers. *International Migration,**47*(2), 3–31. 10.1111/j.1468-2435.2008.00479.x

[CR17] Brijnath B (2009). Familial bonds and boarding passes: understanding caregiving in a transnational context. Identities.

[CR18] Baumeister RF, Stillwell AM, Heatherton TF (1994). Guilt: an Interpersonal Approach. Psychological Bulletin.

[CR19] Canadian Bureau for International Education (2018). *International Students in Canada 2018*: *Canada’s Performance and Potential in International Education*. https://cbie.ca/wp-content/uploads/2018/04/Infographic-inbound-EN.pdf. Accessed 20 Feb 2022

[CR20] Canadian Standard Association (2018). *Search results*. B701 Package Carer-inclusive and accommodating organisations. Retrieved from. https://www.csagroup.org/store/search-results/?search=all~~carer-inclusive+and+accommodating+organizations+standard%5D. Accessed 03 Mar 2019

[CR21] Clarke A, Skuterud M (2013). Why do immigrant workers in Australia perform better than those in Canada? Is it the immigrants or their labour markets?. Canadian Journal of Economics/Revue Canadienne D’économique.

[CR22] Castañeda H, Holmes SM, Madrigal DS, Young MEDT, Beyeler N, Quesada J (2015). Immigration as a social determinant of health. Annual Review of Public Health.

[CR23] Crossman, E., Choi, Y., & Hou, F. (2021). *International students as a source of labour supply: The growing number of international students and their changing sociodemographic characteristics*. Retrieved from https://www150.statcan.gc.ca/n1/pub/36-28-0001/2021007/article/00005-eng.htm

[CR24] Das Gupta, T. (2020). *Inquiry into coronavirus nursing home deaths needs to include discussion of workers and Race*. The Conversation. Retrieved from https://theconversation.com/inquiry-into-coronavirus-nursing-home-deaths-needs-to-include-discussion-of-workers-and-race-139017

[CR25] Dekker R, Engbersen G (2014). How social media transform migrant networks and facilitate migration. Global Networks.

[CR26] Dhar VE (2011). Transnational caregiving: part 1, caring for family relations across nations. Care Management Journals.

[CR27] Dhar VE (2011). Transnational caregiving: part 2, caring for family relations across nations. Care Management Journals.

[CR28] Donovan R, Williams AM (2014). Care-giving as a canadian vietnamese tradition: “It’s like eating, you just do it”. Health & Social Care in the Community.

[CR29] Edmonston B (2016). Canada’s immigration trend and patterns. Canadian Studies in Population.

[CR30] El-Assal, K. (2020). *642,000 international students: Canada now ranks 3rd globally in foreign student attraction*. CIC News. Retrieved from https://www.cicnews.com/2020/02/642000-international-students-canada-now-ranks-3rd-globally-in-foreign-student-attraction-0213763.html#gs.o9mdmd

[CR31] Fast, J., Dosman, D., Lero, D., & Lucas, S. (2013). (rep.). *The intersection of caregiving and employment across the life course: Final report*. Centre for Families, Work & Well-Being, University of Guelph.

[CR32] Ferazzoli, M. T., & Walsh, J. (2021, December 10). *Covid travel restrictions have created new borders for migrants who want to visit home*. The Conversation. Retrieved from https://theconversation.com/covid-travel-restrictions-have-created-new-borders-for-migrants-who-want-to-visit-home-171461

[CR33] Ferrer I (2015). Examining the disjunctures between policy and care in Canada’s parent and grandparent supervisa. International Journal of Migration Health and Social Care.

[CR34] Government of Canada (2019). *2018 Annual Report to Parliament on Immigration*. https://www.canada.ca/en/immigration-refugees-citizenship/corporate/publications-manuals/annual-report-parliament-immigration-2018/report.html

[CR35] Gui T, Koropeckyj-Cox T (2016). “I am the only child of my parents:” perspectives on future elder care for parents among chinese only-children living overseas. Journal of Cross-Cultural Gerontology.

[CR36] Guo S (2013). The changing face of work and learning in the context of immigration: the canadian experience. Journal of Education and Work.

[CR37] Heymann J, Flores-Macias F, Hayes JA, Kennedy M, Lahaie C, Earle A (2009). The impact of migration on the well-being of transnational families: new data from sending communities in Mexico. Community Work & Family.

[CR38] Ho B, Friedland J, Rappolt S, Noh S (2003). Caregiving for relatives with alzheimer’s disease: feelings of chinese-canadian women. Journal of Aging Studies.

[CR39] Hochschild AR, Hutton W, Giddens A (2000). ’global care chains and emotional surplus value’. On the edge: living with global capitalism.

[CR40] Ilagan C, Akbar Z, Sethi B, Williams A (2020). Use of photovoice methods in research on informal caring: a scoping review of the literature. Journal of Human Health Research.

[CR41] Ireson R, Sethi B, Williams A (2016). Availability of caregiver-friendly workplace policies (CFWPs): an international scoping review. Health & Social Care in the Community.

[CR42] Kodwo-Nyameazea, Y., & Nguyen, P. V. (2008). Immigrants and long-distance elder care: An exploratory study. *Ageing International*, *32*, 279–297.

[CR43] Lahaie C, Hayes J, Piper T, Heymann J (2009). Work and family divided across borders: the impact of parental migration on mexican children in transnational families. Community Work & Family.

[CR44] Lai DW (2007). Cultural predictors of caregiving burden of chinese-canadian family caregivers. Canadian Journal on Aging/La Revue Canadienne Du Vieillissement.

[CR45] Lai DW (2008). Intention of use of long-term care facilities and home support services by chinese-canadian family caregivers. Social Work in Health Care.

[CR46] Lai DW, Leonenko W (2007). Effects of caregiving on employment and economic costs of chinese family caregivers in Canada. Journal of Family and Economic Issues.

[CR47] Lee YS, Chaudhuri A, Yoo GJ (2015). Caring from afar: asian H1B migrant workers and ageing parents. Journal of Cross-Cultural Gerontology.

[CR48] Levitt, P. (2019). *Transnational migrants: When “home” means more than one country*. https://www.migrationpolicy.org/article/transnational-migrants-when-home-means-more-one-country

[CR49] Lewis, H. (2001). Universal mother: Transnational migration and the human rights of black women in the Americas. *The Journal of Gender, Race, and Justice, 5*(1), 198–231.

[CR50] Lorenz F, Whittaker L, Tazzeo J, Williams A (2021). Availability of caregiver-friendly workplace policies: an international scoping review follow-up study. International Journal of Workplace Health Management.

[CR51] Lunt. (2009). Older people within transnational families: the social policy implications. *International Journal of Social Welfare, 18*(3), 243–251. 10.1111/j.1468-2397.2008.00600.x

[CR52] Madianou M (2014). Polymedia communication and mediatized migration: an ethnographic approach. Mediatization of Communication.

[CR53] Madianou M (2016). Ambient co-presence: transnational family practices in polymedia environments. Global Networks.

[CR54] Madianou M, Miller D (2013). Polymedia: towards a new theory of digital media in interpersonal communication. International Journal of Cultural Studies.

[CR55] Miyawaki, C. E., & Hooyman, N. R. (2021). A systematic review of the literature on transnational caregiving: immigrant adult children to ageing parents in home country. *Journal of Family Studies*, 1–18. 10.1080/13229400.2021.1908908

[CR56] Mohsin, M. (2022). *10 social media statistics you need to know in 2021*. Oberlo. Retrieved from https://www.oberlo.in/blog/social-media-marketing-statistics

[CR57] Morse, J. M. (1991). Strategies for sampling. In J. M. Morse (Ed.), *Qualitative nursing research: a contemporary dialogue* (pp. 127–145). Sage Publications, Inc. 10.4135/9781483349015.n16

[CR58] Morse, J. M., & Field, P. A. (1995). *Qualitative research methods for health professionals*. Sage Publications.

[CR59] Neuendorf, K. A. (2016). *The Content Analysis Guidebook* (2nd ed.). SAGE. Retrieved from https://us.sagepub.com/en-us/nam/the-content-analysis-guidebook/book234078#contents

[CR60] Neysmith SM, Zhou YR (2013). Mapping another dimension of a feminist ethics of care: family-based transnational care. IJFAB: International Journal of Feminist Approaches to Bioethics.

[CR61] Park, M. (2012). Filial piety and parental responsibility: an interpretive phenomenological study of family caregiving for a person with mental illness among korean immigrants. *BMC Nursing*, *11*(1). 10.1186/1472-6955-11-28 10.1186/1472-6955-11-28 PMC3554497 23256876

[CR62] Peters I, Wilson J (2017). The business case for employers supporting carers: reflecting on a UK model. International Journal of Care and Caring.

[CR63] Schmucker, C. M., Blümle, A., Schell, L. K., Schwarzer, G., Oeller, P., Cabrera, L., von Elm, E., Briel, M., & Meerpohl, J. J. (2017). Systematic review finds that study data not published in full text articles have unclear impact on meta-analyses results in medical research. *PLoS One*, *12*(4). 10.1371/journal.pone.0176210 10.1371/journal.pone.0176210 PMC5404772 28441452

[CR64] Şenyürekli AR, Detzner DF (2008). Intergenerational relationships in a transnational context: the case of turkish families. Family Relations.

[CR65] Sethi, B. (2014). *Intersectional exposures: Exploring the health effect of employment with Kaajal immigrant/refugee women in Grand Erie through photovoice* (dissertation). Retrieved from https://scholars.wlu.ca/etd/1659/

[CR66] Sethi B (2016). Using the eye of the camera to bare racism: a photovoice project. Aotearoa New Zealand Social Work.

[CR67] Sethi, B. (2020). Personal support workers are the backbone of health care but the bottom of the power structure. Retrieved from https://theconversation.com/personal-support-workers-are-the-backbone-of-health-care-but-the-bottom-of-the-power-structure-144648

[CR68] Sethi, B. (2022). Negotiating culture, geographical distance, and employment: The lived experiences of transnational carer employees, S*pecial Issue Proposal for Wellbeing, Space, and Society* (3, pp. 1–8). 10.1016/j.wss.2022.100083

[CR69] Sethi B, Williams A (2016). Microaggressions of caregiver employees: what has social work got to do with it?. Diversity and Equality in Health and Care.

[CR70] Sethi B, Williams A (2017). Working multiple shifts: intersecting dynamics of gender, employment status, and immigrant status in the experiences of immigrant caregiver-employees in urban-rural Canada. Frontiers in Women’s Health.

[CR71] Sethi, B., & Williams, A. (2021). Balancing the Business of Health Care with Staff Care. Research Brief, Stream A, Immigrant/Refugee Employees and Caregiving: A Case Study of Caregiver-Employees in Grand Erie, Ontario. *Gender Health and Caregiver Friendly Workplaces* Retrieved from https://ghw.mcmaster.ca/app/uploads/2019/11/FINAL_ResearchbriefEmployers_Study6_June18.pdf

[CR72] Sethi B, Williams A, Desjardins E, Zhu H, Shen E (2018). Family conflict and future concerns: Opportunities for social workers to better support chinese immigrant caregiver employees. Journal of Gerontological Social Work.

[CR73] Sethi B, Williams A, Ireson R (2017). Supporting caregiver employees: managers’ perspective in Canada. International Journal of Workplace Health Management.

[CR74] Sethi B, Williams A, Zhu H, Shen E, Ireson R (2017). Father is the sky. Mother is the earth: the influence of filial piety in the caregiving experiences of Mandarin-speaking Chinese caregiver-employees in Southern Ontario, Canada. Journal of Diversity and Equality in Health and Care.

[CR75] Sethi B, Vito R, Ongbanouekeni V (2021). Organizational culture, diversity, and employees’ health in Social/Human Services: a systematic review. International Health Trends and Perspectives.

[CR76] Singh S, Cabraal A, Robertson S (2010). Remittances as a currency of care: a focus on ‘twice migrants’ among the indian diaspora in Australia. Journal of Comparative Family Studies.

[CR77] Spitzer, D., Neufeld, A., Harrison, M., Hughes, K., & Stewart, M. (2006). Caregiving in transnational context: “My wings have been cut; where can I fly?“. *Global Dimensions of Gender and Carework*, 176–192. 10.1515/9781503625723-016

[CR78] Tung C (2000). The cost of caring: the social reproductive labor of Filipina live-in home health caregivers. Frontiers: A Journal of Women Studies.

[CR79] Turcotte, M., & Savage, K. (2020). The contribution of immigrants and population groups designated as visible minorities to nurse aide, orderly and patient service associate occupations. Retrieved from https://www150.statcan.gc.ca/n1/pub/45-28-0001/2020001/article/00036-eng.htm

[CR80] Vuksan M, Williams AM, Crooks VA (2012). Family friendly policies: accommodating end-of‐life caregivers in workplaces. International Journal of Workplace Health Management.

[CR81] Vuksan M, Williams AM, Crooks VA (2012). The workplace perspective on supporting family caregivers at end of life: evaluating a new canadian social program. Community Work & Family.

[CR82] Wang L, Williams A, Kitchen P (2018). Health of caregiver-employees in Canada. International Journal of Workplace Health Management.

[CR83] Westhues A, Ochocka J, Jacobson N, Simich L, Maiter S, Janzen R, Fleras A (2008). Developing theory from complexity: reflections on a collaborative mixed method participatory action research study. Qualitative Health Research.

[CR84] Wilding R, Baldassar L (2018). Ageing, migration and new media: the significance of transnational care. Journal of Sociology.

[CR85] Williams A, Cortissoz C, Yazdani A (2018). Better support for carer-employees in Canada: the development of standardised guidelines for the workplace. International Journal of Care and Caring.

[CR86] Zagrodney K, Saks M (2017). Personal support workers in Canada: the new precariat?. Healthcare Policy | Politiques De Santé.

[CR87] Zhou Y (2013). Toward transnational care interdependence: rethinking the relationships between care, immigration and Social Policy. Global Social Policy.

